# Effects of *Ulmus macrocarpa* Extract and Catechin 7-O-*β*-D-apiofuranoside on Muscle Loss and Muscle Atrophy in C2C12 Murine Skeletal Muscle Cells

**DOI:** 10.3390/cimb46080491

**Published:** 2024-08-01

**Authors:** Min Seok Kim, Sunmin Park, Yeeun Kwon, TaeHee Kim, Chan Ho Lee, HyeonDu Jang, Eun Ji Kim, Jae In Jung, Sangil Min, Kwang-Hyun Park, Sun Eun Choi

**Affiliations:** 1 Dr. Oregonin Inc., #802 Bodeum Hall, Kangwondaehakgil 1, Chuncheon 24341, Republic of Korea; ms23217@naver.com (M.S.K.); dpkssm0929@naver.com (S.P.); kye0519@naver.com (Y.K.); kth02120@naver.com (T.K.); 2 Department of Forest Biomaterials Engineering, Kangwon National University, Chuncheon 24341, Republic of Korea; lgh4107@naver.com (C.H.L.); wkdgusen98@naver.com (H.J.); 3 Industry Coupled Cooperation Center for Bio Healthcare Materials, Hallym University, Chuncheon 24252, Republic of Korea; myej4@hallym.ac.kr (E.J.K.); jungahoo@hallym.ac.kr (J.I.J.); 4 Division of Transplantation and Vascular Surgery, Department of Surgery, Seoul National University Hospital, Seoul 03080, Republic of Korea; surgeonmsi@gmail.com; 5 Department of Emergency Medical Rescue, Nambu University, Gwangju 62271, Republic of Korea; pkh15129@gmail.com

**Keywords:** *Ulmus macrocarpa*, Catechin 7-O-*β*-D apiofuranoside, apoptosis, muscle atrophy, sarcopenia

## Abstract

Muscle atrophy is known to be one of the symptoms leading to sarcopenia, which significantly impacts the quality of life, mortality, and morbidity. Therefore, the development of therapeutics for muscle atrophy is essential. This study focuses on addressing muscle loss and atrophy using *Ulmus macrocarpa* extract and its marker compound, catechin 7-O-*β*-D-apiofuranoside, by investigating their effects on biomarkers associated with muscle cell apoptosis. Additionally, protein and gene expression in a muscle atrophy model were examined using Western blotting and RT-PCR. *Ulmus macrocarpa* has been used as food or medicine due to its safety, including its roots, barks, and fruit. Catechin 7-O-*β*-D apiofuranoside is an indicator substance of plants of the *Ulmus* genus and has been reported to have various effects such as antioxidant and anti-inflammatory effects. The experimental results demonstrated that catechin glycoside and *Ulmus macrocarpa* extract decreased the expression of the muscle-degradation-related proteins Atrogin-1 and Muscle RING-Finger protein-1 (MuRF1) while increasing the expression of the muscle-synthesis-related proteins Myoblast determination (MyoD) and Myogenin. Gene expression confirmation experiments validated a decrease in the expression of Atrogin and MuRF1 mRNA and an increase in the expression of MyoD and Myogenin mRNA. Furthermore, an examination of muscle protein expression associated with the protein kinase B (Akt)/forkhead box O (FoxO) signaling pathway confirmed a decrease in the expression of FoxO, a regulator of muscle protein degradation. These results confirm the potential of *Ulmus macrocarpa* extract to inhibit muscle apoptosis, prevent muscle decomposition, and promote the development of functional materials for muscle synthesis, health-functional foods, and natural-product-derived medicines.

## 1. Introduction

Aging is a significant factor in demographic changes worldwide. With advances in medical science leading to increased average lifespans and declining birth rates resulting in a growing elderly population, there is heightened interest in issues related to aging, such as anti-aging and disease prevention [1]. Muscle atrophy, known to be one of the symptoms leading to sarcopenia, involves the loss of muscle mass, weakness, fatigue, and reduced muscle contraction activity. These conditions contribute to the worsening of chronic diseases such as chronic heart failure, chronic kidney disease, and cancer. Current treatment methods for muscle atrophy primarily include exercise and nutritional supplements [2]. However, exercise can be limited for the elderly and bedridden patients. Thus, there is a pressing need for the development of safe and harmless therapeutic agents to prevent muscle atrophy.

*Ulmus macrocarpa* belongs to the Ulmaceae family, and is known in traditional Korean medicine as Yubaekpi (楡白皮), with the dried root bark known as Yugeunpi (楡根皮). It is considered non-toxic and has been traditionally used for conditions such as eczema, arthritis, and inflammation [3]. In folk medicine, it has also been utilized to treat gastrointestinal disorders, edema, and cancer [4]. Plants of the *Ulmus* genus contain (-)-catechin, triterpene, and neolignan glycoside, and these are known to exert their effects through their metabolites after absorption and metabolism. [5,6,7,8]. In a recent study conducted by our research team, various parts (leaves, stems, and bark) of four indigenous *Ulmus* genus plants were subjected to phytochemical analyses using HPLC and NMR. Through this analysis, catechin 7-O-*β*-D-apiofuranoside was identified as a standard compound that can be used to determine the chemotaxonomy of the *Ulmus* genus [6]. Numerous previous studies have also confirmed that the characteristic compound of the *Ulmus* genus is catechin 7-O-*β*-D-apiofuranoside [9,10]. This compound has been reported to exhibit various and remarkable biological activities, including antioxidant effects [11], anti-inflammatory effects [5], immunomodulatory effects [12], and the regulation of apoptosis in human papilla cells [13]. Therefore, our goal is to develop a therapeutic agent that utilizes natural compounds derived from *Ulmus macrocarpa* for the prevention of muscle loss and atrophy.

In this study, we investigated biomarkers related to the inhibition of muscle cell apoptosis in an *Ulmus* macrocarpa extract and its active component, catechin 7-O-*β*-D-apiofuranoside. Additionally, we employed a dexamethasone-induced muscle cell model to verify their anti-atrophy effects and performed a detailed validation of the protein and mRNA expression of muscle-synthesis- and atrophy-related biomarkers, such as atrogin1, MuRF1, Myogenin, and MyoD. Our research team conducted an in vitro mechanism study that covered four distinct categories—inhibiting oxidative stress, anti-apoptosis effects, promoting muscle protein synthesis, and inhibiting muscle protein degradation—to explore the mechanisms through which *Ulmus macrocarpa* prevents muscle loss and atrophy and its effectiveness.

## 2. Materials and Methods

### 2.1. Plant Extract Materials

This study was conducted using an *Ulmus macrocarpa* extract and isolated and purified catechin 7-O-*β*-D apiofuranoside from the extract. The *Ulmus macrocarpa* bark used in this study was purchased from Seoul Yakryeong Market and was used after it was certified by Professor Choi (Department of Forest Biomaterials Engineering, Kangwon National University). The bark was cleaned and washed to remove impurities and used as an experimental material. A sample of the *Ulmus macrocarpa* extract (UME-2023-04) is stored at the Department of Forest Biomaterials Engineering, Kangwon National University.

### 2.2. Pilot-Scale Extraction and Solvent Fractionation of Bark of Ulmus macrocarpa

In order to determine the optimal yield conditions and the standard component content of *Ulmus macrocarpa* at the lab scale stage, *Ulmus macrocarpa* was extracted at different edible ethanol ratios (25%, 50%, and 100%) and extraction times (4 h and 8 h). In the semi-pilot scale stage, dextrin, a forming agent, was added at concentrations of 0, 5, 10, 25, and 50% to the *Ulmus macrocarpa* extract. Subsequently, in the pilot scale stage, 300 kg of *Ulmus macrocarpa* raw material was concentrated under a 50% edible ethanol concentration and 80 °C, resulting in the acquisition of 60 kg of *Ulmus macrocarpa* concentrate (yield 20%, Lot. No. DJTT-22969). From this, 31 kg of *Ulmus macrocarpa* concentrate was freeze-dried, recovering 14.105 kg of powder (yield 45.5%). To this powder, 10% dextrin (1.411 kg) was added, resulting in the obtainment of 15.516 kg of *Ulmus macrocarpa* extract powder (UME, Lot. No. DJTT-06789). The extraction process was conducted by DanjoungBio Co., Ltd. (Wonju, Republic of Korea).

### 2.3. Separation and Purification of Catechin Glycoside

**Figure 1:**
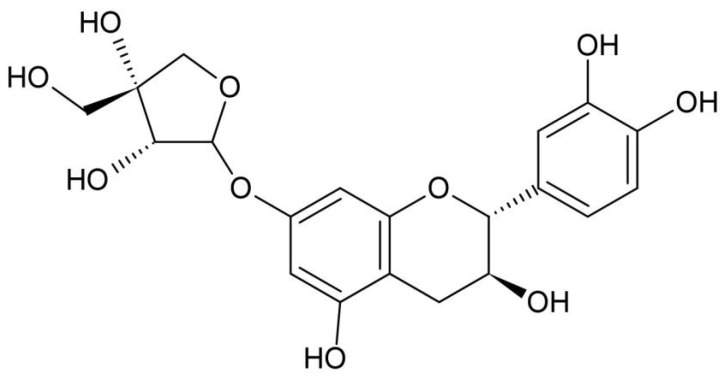
The structure of catechin 7-O-*β*-D apiofuranoside.

The purification and isolation of catechin 7-O-*β*-D-apiofuranoside (CAG) were performed using the method detailed in Kwon et al., 2022 [13] (Figure 1). 

Catechin 7-O-*β*-D-apiofuranoside (**1**) Brown amorphous powder, Negative LC-MS/MS: *m*/*z* 422.38 [M − H]^−^

^1^H-NMR (400 MHz, MeOH-*d*_4_): 6.82 (H-2′, 1H, d, J = 1.2 Hz), 6.76 (H-5′, 1H, d, J = 7.6 z), 6.72 (H-6′, 1H, dd *J* = 2, 8 Hz), 6.12 (H-8, 1H, d, *J* = 2.4 Hz), 6.07 (H-6, 1H, d, *J* = 2.4 Hz), 5.47 (H-1″, 1H, d, *J* = 3.2 Hz), 4.58 (H-2, 1H, d, *J* = 7.6 Hz), 4.13 (H-2″, 1H, d, *J* = 3.2 Hz), 4.06 (H-4a″, 1H, d, *J* = 10 Hz), 3.99 (H-3, 1H, m), 3.84 (H-4b″, 1H, d, *J* = 10 Hz), 3.60 (H-5″, 2H, m), 2.82 (H-4a, 1H, dd, *J* = 5.6, 16.8 Hz), and 2.56 (H-4b, 1H, dd, *J* = 8, 16.4 Hz) [6].

^13^C-NMR (100 MHz, MeOH-*d*_4_): 158.3 (C-7), 157.7 (C-5), 157.0 (C-9), 146.4 (C-4′), 146.4 (C-3′), 132.3 (C-1′), 120.1 (C-6′), 116.2 (C-5′), 115.3 (C-2′), 108.8 (C-1″), 103.4 (C-10), 97.0 (C-8), 97.4 (C-6), 83.1 (C-2), 80.4 (C-3″), 78.4 (C-2″), 75.6 (C-4″), 68.8 (C-3), 65.1 (C-5″), and 28.6 (C-4) [6].

### 2.4. Quantitative Chromatographic Analysis of Ulmus macrocarpa Extract Powder (UME)

**Figure 2:**
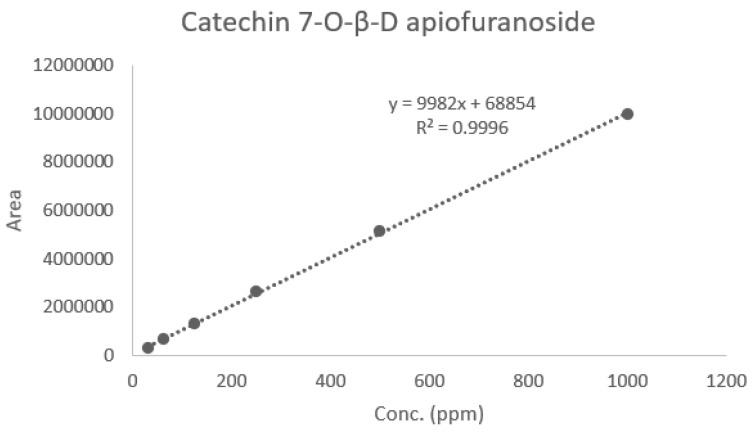
Calibration curve and equation of catechin 7-O-*β*-D apiofuranoside (1000 μg/mL, 500 μg/mL, 250 μg/mL, 125 μg/mL, 62.5 μg/mL, and 31.25 μg/mL).

**Figure 3:**
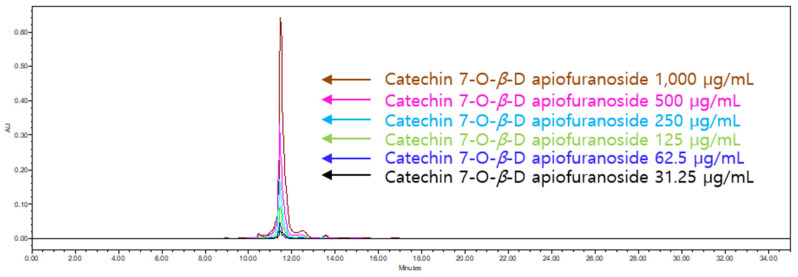
HPLC chromatogram of catechin 7-O-*β*-D apiofuranoside (1000 μg/mL, 500 μg/mL, 250 μg/mL, 125 μg/mL, 62.5 μg/mL, and 31.25 μg/mL of CAG).

**Figure 4:**
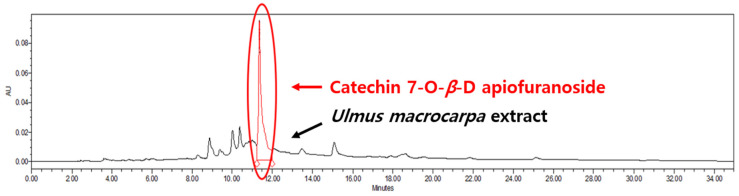
HPLC chromatogram of *Ulmus macrocarpa* extract powder (1000 μg/mL).

An HPLC analysis of *Ulmus macrocarpa* extract powder (UME) was performed using a Waters 2695 (Waters, Milford, MA, USA). Its column conditions were used in combination with a SkyPak C18 column (5 μm) and Phenomenex KJ0-4282 guard column. The injected volume was 20 μL and the wavelength was 280 nm. The analysis was conducted at a flow rate of 1 mL/min for 35 min using 0.9% acetic acid in water and acetonitrile as the mobile phases. The relationship between the concentration and the peak area was measured using the minimum square method (R^2^ value). A standard calibration curve and the equation of that curve were obtained from six concentrations of catechin 7-O-*β*-D apiofuranoside (CAG) (Figure 2). This resulted in Y = 9982x + 68,854 (R^2^ = 0.999) increments of CAG, as shown in Figure 2. The calibration curve has good linearity (correlation coefficient ≥ 0.999). A chromatogram of CAG is shown in Figure 3, and the average content of the UME was calculated as 133.36 ± 0.27 μg/mL using the formula above (Figure 4).

### 2.5. Cell Culture and Treatments

C2C12 cells, myoblasts derived from mouse skeletal muscle, were purchased from the American Type Culture Collection (ATCC). C2C12 cells were cultured in Dulbecco’s Modified Eagle Medium (DMEM) supplemented with 10% fetal bovine serum (FBS), 100 units/mL of penicillin, and 100 μg/mL streptomycin and incubated at 37 °C in a humidified CO_2_ incubator (5% CO_2_/95% air).

#### 2.5.1. Apoptosis Induction and Treatment

C2C12 myoblasts were exposed to 100 μM of H_2_O_2_ to induce apoptosis. To investigate the effect of UME and CAG on H_2_O_2_-induced apoptosis in C2C12 myoblasts, cells were plated in multi-well plates and incubated for 24 h. Afterwards, C2C12 myoblasts were treated with 100 μM of H_2_O_2_ with/without different concentrations of UME or CAG, as indicated [14].

#### 2.5.2. Differentiation Induction and Treatment

To determine the effect of UME and CAG on cell viability in myotubes in the presence of dexamethasone (Sigma-Aldrich, Darmstadt, Germany), C2C12 cells were plated in 24-well plates at a density of 5 × 10^4^ cells/well and differentiated into myotubes for 4 days, as described above. After the induction of differentiation, the myotubes were treated with 5 μM of dexamethasone and various concentrations of UME or CAG for 24 h. 

### 2.6. Effects of Ulmus Macrocarpa Extract Powder (UME) and Catechin 7-O-β-D-apiofuranoside (CAG) on Muscle Apoptosis Biomarkers

#### 2.6.1. Evaluation of Apoptosis in H_2_O_2_-Induced Myoblasts

To evaluate the effects of the test substances on H_2_O_2_-induced apoptosis in myoblasts, C2C12 cells were seeded into 24-well plates at a density of 5 × 10^4^ cells/well and cultured for 24 h. After the initial 24 h culture period, the cells were treated with 100 μM of H_2_O_2_ to induce myoblast damage. To investigate the protective effects of the test substances against myoblast damage, five different test substances were administered at various concentrations in conjunction with 100 μM of H_2_O_2_, and the cells were cultured for an additional 48 h. The extent of the apoptosis in myoblasts was measured using a Cellular DNA Fragmentation ELISA kit (Sigma-Aldrich), which detects 5′-Bromo-2′-deoxy-uridine (BrdU)-labeled DNA, following the manufacturer’s protocol.

#### 2.6.2. Western Blot Analysis

C2C12 myoblasts were plated and treated with UME or CAG in the presence of 100 μM of H_2_O_2_ for 24 h, as described above. After that, the cells were lysed and Western blot analyses were conducted as described previously [15]. Antibodies against Bax, Bcl-2, cleaved caspase-3, cleaved PARP, and β-actin (Cell Signaling Technology, Beverly, MA, USA) were used in this analysis. The protein bands were developed using the Luminata^TM^ Forte Western HRP Substrate (Millipore, Billerica, MA, USA) and captured and analyzed for their intensity using an ImageQuant^TM^ LAS 500 imaging system (GE Healthcare Bio-Sciences AB, Uppsala, Sweden). Their relative protein expressions were normalized to β-actin. 

### 2.7. Effects of Ulmus Macrocarpa Extract (UME) and Catechin 7-O-β-D apiofuranoside (CAG) on Muscle-Synthesis- and Muscle-Degradation-Related Proteins and Gene Expression

#### 2.7.1. Measurement of Myotube Diameter 

C2C12 cells were plated in 24-well plates at a density of 5 × 10^4^ cells/well, differentiated into myotubes for 4 days, and treated with UME or CAG and 5 μM of dexamethasone for 24 h, as described above. After that, the cells were fixed with 4% paraformaldehyde, permeabilized with 0.1% Triton X-100, and blocked with 5% BSA. The cells were immunostained with MYH7 antibody and Alexa Fluor 594 labeled goat anti-mouse IgG antibody. DAPI (Sigma-Aldrich) was applied as a counterstain for the nuclei. Fluorescent cell images (6 images per group) were captured using microscopy (AxioImager, Carl Zeiss, Jena, Germany) at 20× magnification. Ten myotubes per image were chosen on a random basis from each micrograph. The thickest portion of each myotube was analyzed for its myotube diameter using ImageJ software (National Institutes of Health, Bethesda, MD, USA, Version 1.54).

#### 2.7.2. Real-Time Reverse Transcription Polymerase Chain Reaction (RT-PCR) Analysis

C2C12 cells were plated, differentiated into myotubes for 4 days, and treated with UME or CAG and 5 μM of dexamethasone for 24 h, as described above. Total RNA was extracted using an RNeasy^®^ Plus Mini kit (Qiagen, Valencia, CA, USA) according to the manufacturer’s instructions. The extracted total RNA was quantified using a micro-volume spectrophotometer (BioSpec-nano, Shimadzu, Kyoto, Japan). Reverse transcription from 2 μg of total RNA was performed using a HyperScript^TM^ RT master mix kit (GeneAll Biotechnology). A real-time PCR was performed using a QuantiNova SYBR Green PCR kit (Qiagen) on a Rotor-gene 300 PCR (Corbett Research, Mortlake, Australia), as described previously [16]. The sequences of the primers used in this PCR are shown in Table 1. Data analysis was conducted using the Rotor-Gene 6000 series System Soft program version 6 (Corbett). The relative expression levels of the target mRNA were normalized to that of the housekeeping protein glyceraldehyde 3-phosphate dehydrogenase (GAPDH) [10].

#### 2.7.3. Western Blot Analysis

C2C12 myoblasts were plated, differentiated into myotubes for 4 days, and treated with UME and 5 μM of dexamethasone for 24 h, as described above. After that, the cells were lysed and Western blot analyses were conducted as described previously [16]. Antibodies against Atrogin-1, MuRF-1 (Santa Cruz, Santa Cruz, CA, USA), MyoD1, Myogenin (Abcam, Cambridge, MA, USA), phospho-Akt (Ser473), Akt, phospho-mTOR (Ser253), mTOR, phospho-FoxO1 (Ser256), FoxO1, phospho-FoxO3 (Ser253), FoxO3, and β-actin (Cell Signaling Technology) were used in this analysis. The protein bands were developed using the Luminata^TM^ Forte Western HRP Substrate (Millipore, Billerica, MA, USA) and then captured and analyzed for their intensity using the ImageQuant^TM^ LAS 500 imaging system (GE Healthcare Bio-Sciences AB, Uppsala, Sweden). Their relative protein expressions were normalized to β-actin. 

### 2.8. Statistical Analysis

All values from the analyses are expressed as mean ± S.E.M. The collected results were analyzed using the GraphPad Prism 5.0 (GraphPad Software, San Diego, CA, USA) program. Student’s *t*-test and a one-way analysis of variance (ANOVA) were used to compare the differences between the treatment group and the control group. These were judged to be statistically significant only when *p* < 0.05.

## 3. Results

### 3.1. The Impact of UME and CAG on the Viability of C2C12 Cells

#### Measurement of Cell Viability under Normal Conditions

**Figure 5:**
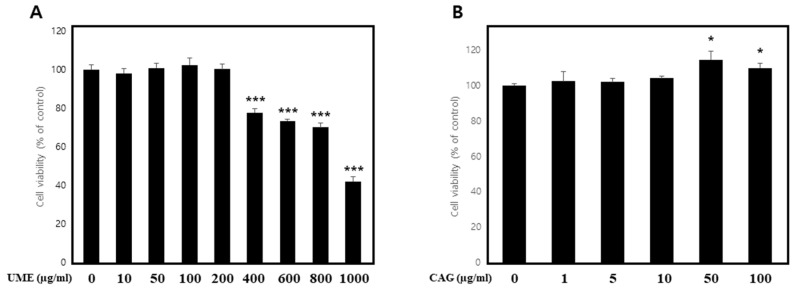
Cytotoxicity of (**A**) *Ulmus macrocarpa* extract (UME) and (**B**) catechin 7-O-*β*-D apiofuranoside (CAG) on C2C12 cells. Cell viability was calculated as described in the Materials and Methods. Values are expressed as mean ± S.E.M. (n = 3). * *p* < 0.05, *** *p* < 0.001 are significantly different from that of the 0 μg/mL group.

To investigate cytotoxicity in C2C12 cells, different concentrations of UME and CAG were added to the cell culture medium and cultured for 48 h, and then an MTT assay was performed. Treatment with UME (Figure 5A) resulted in a decrease in cell viability at concentrations of 400 μg/mL and above, while treatment with CAG (Figure 5B) did not decrease cell viability to 80% or below at any treatment concentration (Figure 5). Based on these results, the highest treatment concentrations for UME and CAG were set to 200 μg/mL and 100 μg/mL, respectively, for further experiments.

### 3.2. Effects of Ulmus Macrocarpa Extract (UME) and Catechin 7-O-β-D Apiofuranoside (CAG) on Muscle Apoptosis Biomarkers

#### 3.2.1. Effects on H_2_O_2_-Induced Apoptosis in Myoblasts

**Figure 6:**
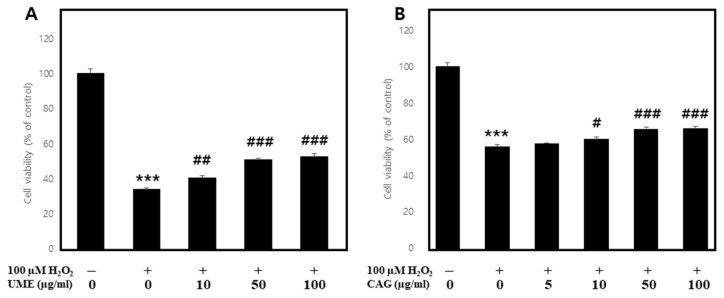
Protective effect of (**A**) *Ulmus macrocarpa* extract (UME) and (**B**) catechin 7-O-*β*-D apiofuranoside (CAG) on cell viability in H_2_O_2_-treated C2C12 myoblasts. Values are expressed as mean ± S.E.M. (n = 3). *** *p* < 0.001 are significantly different from that of the [H_2_O_2_(−)/UME(−)], [H_2_O_2_(−)/CAG(−)] group. ^#^ *p* < 0.05, ^##^ *p* < 0.01, and ^###^ *p* < 0.001 are significantly different from that of the [(+)/(−)] group.

In models of muscle atrophy caused by diabetes, cancer, heart failure, AIDS, and sepsis, skeletal muscle atrophy, which is associated with oxidative stress, is induced [17,18,19,20]. Oxidative stress leads to the generation of reactive oxygen species, activating the ubiquitin pathway. This results in increased protein degradation and decreased myosin expression, and ultimately leads to muscle atrophy [21,22,23]. To investigate the protective effect of UME and CAG against muscle cell damage, oxidative stress was induced in C2C12 cells using H_2_O_2_ and each substance was administered to determine cell viability (Figure 6) [24,25]. When normal cells were treated with 100 μM of H_2_O_2_, the cell viability rate decreased to 34.1 ± 0.9%, and, when treated with UME (Figure 6A), it increased in a concentration-dependent manner, reaching 52.6 ± 1.6% at the highest treatment concentration of 100 μg/mL, about an 18.5% increase. When apoptosis was induced using H_2_O_2_, the concentration-dependent cell viability was significantly decreased when treated with CAG (Figure 6B), increasing by 8.3% at the highest treatment concentration of 100 μg/mL. Accordingly, the protective effects of UME and CAG against H_2_O_2_-induced myoblast cell damage were confirmed.

#### 3.2.2. Effects of Catechin 7-O-*β*-D apiofuranoside on Bax, Bcl-2, Cleaved Caspase-3, and Cleaved PARP Protein Expression

Apoptosis is essential for normal growth and homeostasis in all multicellular organisms and it occurs via two pathways: the intrinsic pathway, via the mitochondria, and the extrinsic pathway. In both pathways, the activation of a family of sequential cysteine proteases, the caspase enzymes, occurs concomitantly [26,27] and continuously [28]. In research to date, the mechanisms through which mitochondria work in the apoptosis pathway can be broadly divided into two types. First, there are mechanisms such as the Bcl-2 family [29], which are muscle proteins that control the apoptosis pathway and protect cells, and Bax [30] and Apaf-1 [31], which are genes that induce apoptosis. Bcl-2 interrupts the release of cytochrome C from the mitochondria or binds to muscle proteins around the mitochondria, preventing apoptosis [27]. On the other hand, Bax facilitates the release of cytochrome C in response to stress such as DNA damage, triggering apoptosis [32]. Second, they allow for the activation of caspases. Caspases, a crucial type of protease involved in various cellular functions, including differentiation and apoptosis, are activated by cytochrome C being released into the cytoplasm. This activates caspases such as caspase-8, caspase-9, and caspase-2, leading to the activation of downstream caspases like caspase-3 and caspase-6. These caspases cleave cellular muscle proteins, inducing apoptosis [33]. Of these, the activation of caspase-3 is particularly crucial as it occurs just before apoptosis [34].

**Figure 7:**
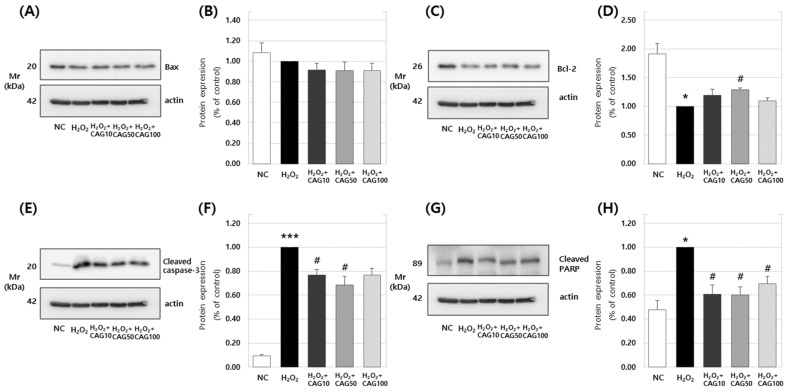
Anti-apoptotic effects of catechin 7-O-*β*-D apiofuranoside (CAG) on H_2_O_2_-induced oxidative damage in C2C12. Western blotting was performed to analyze levels of (**A**,**B**) Bax, (**C**,**D**) Bcl-2, (**E**,**F**) cleaved caspase-3, (**G**,**H**) cleaved PARP, and actin, as described in Materials and Methods. Values are expressed as mean ± S.E.M. (n = 3). * *p* < 0.05, *** *p* < 0.001 are significantly different from that of the untreated H_2_O_2_ group. ^#^ *p* < 0.05 are significantly different from that of the H_2_O_2_-treated group.

We investigated the effect of CAG on apoptosis in H_2_O_2_-induced C2C12 cells (Figure 7). The results showed that the expression of Bax (Figure 7A,B) was not significantly different between the group without an H_2_O_2_ treatment and groups treated with varying concentrations of CAG after H_2_O_2_ exposure. However, the expression of Bcl-2 (Figure 7C,D) significantly decreased when treated with H_2_O_2_, and particularly at a concentration of 50 μg/mL, where there was a significant increase in Bcl-2 protein expression to 1.29 ± 0.04.

In the H_2_O_2_-treated control group, the expression of cleaved caspase-3 (Figure 7E,F) significantly increased. However, when treated with CAG at concentrations of 10 μg/mL and 50 μg/mL, its expression decreased significantly to 0.77 ± 0.05 and 0.69 ± 0.07, respectively. Similarly, the expression of cleaved PARP (Figure 7G,H) significantly increased in the H_2_O_2_-treated control group. However, when treated with 10–50 μg/mL of CAG, the expression of cleaved PARP decreased significantly at all concentrations, to 0.61 ± 0.08, 0.60 ± 0.07, and 0.69 ± 0.06, respectively. This demonstrates that CAG effectively has an inhibitory effect on muscle apoptosis.

### 3.3. Effects of UME and CAG on Muscle-Synthesis- and Muscle-Degradation-Related Proteins and Gene Expression

#### 3.3.1. Impact of Dexamethasone-Induced Myotube Damage

Dexamethasone (DEX) is a representative glucocorticoid that causes the degradation of skeletal muscle and, based on this, it is widely used to induce muscle cell atrophy in in vitro systems [35,36,37,38]. Previous studies have reported that dexamethasone increases protein degradation through the ubiquitin–proteasome pathway and regulates the expression of related genes [39,40,41].

**Figure 8:**
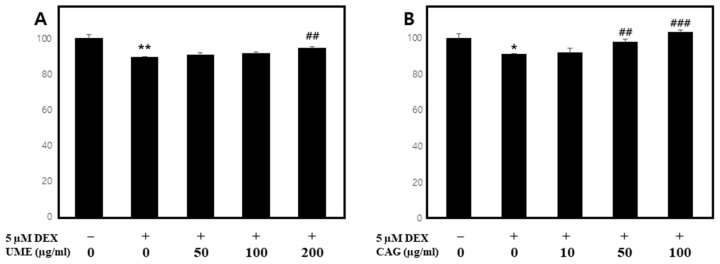
Protective effect of (**A**) *Ulmus macrocarpa* extract (UME) and (**B**) catechin 7-O-*β*-D apiofuranoside (CAG) on cell viability in DEX-treated C2C12 myotubes. Values are expressed as mean ± S.E.M. (n = 3). * *p* < 0.05, ** *p* < 0.01 are significantly different from that of the 0 μg/mL group. ^##^ *p* < 0.01, ^###^ *p* < 0.001 are significantly different from that of the [(+)/(−)] group.

To investigate the effects of UME and CAG on myocyte damage, C2C12 cells were treated with dexamethasone to induce myocyte atrophy, and the viability rate of the myotube cells was then confirmed via treatments with each substance. When the C2C12 cells were treated with dexamethasone (Figure 8), their cell viability decreased to 89.3 ± 0.7%, while treatments with UME (Figure 8A) or CAG (Figure 8B) led to a concentration-dependent increase in cell viability. In particular, treatment with the highest concentration of UME (200 μg/mL) resulted in an increase in cell viability to 94.5 ± 1.0%, while CAG led to an increase in cell viability at concentrations of 50 μg/mL or higher. This confirmed that UME and CAG have a protective effect on dexamethasone-induced myotube damage.

#### 3.3.2. Effect of UME and CAG on Dexamethasone-Induced Myotube Atrophy

**Figure 9:**
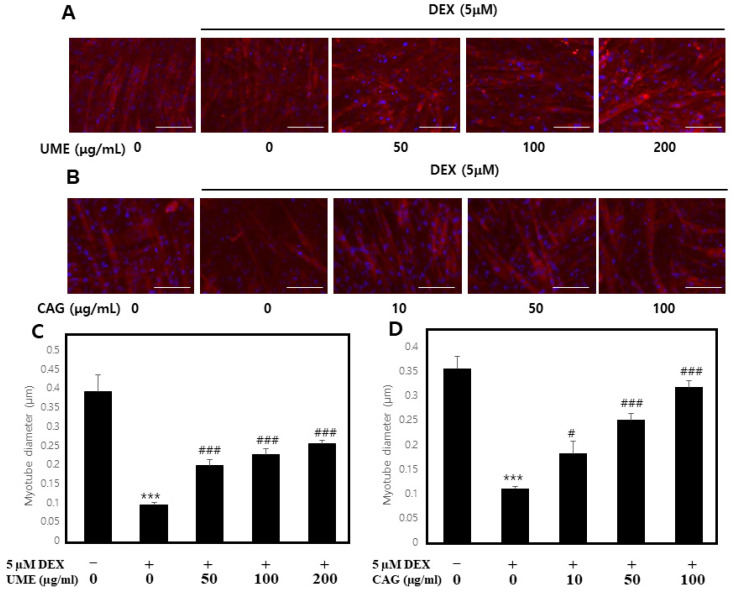
Effects of (**A**,**C**) *Ulmus macrocarpa* extract (UME) and (**B**,**D**) catechin 7-O-*β*-D apiofuranoside (CAG) on dexamethasone-induced muscle atrophy in C2C12 cells. Values are expressed as mean ± S.E.M. (n = 3). Scale bar is 100 μm. *** *p* < 0.001 are significantly different from that of the [(−)/(−)] group. ^#^ *p* < 0.05, ^###^ *p* < 0.001 are significantly different from that of the [(+)/(−)] group.

To investigate the effects of UME and CAG on the dexamethasone-induced atrophy of myotubes, the cells were cultured with UME and CAG treatments, and then the diameter of the myotube cells was measured (Figure 9). When treated with 5 μM of dexamethasone, the diameter of the myocytes was significantly reduced; their diameter decreased to 0.09~0.11 μm compared to the diameter of 0.36~0.39 μm observed in the control group. Subsequently, when 50, 100, and 200 μg/mL of the UME (Figure 9A) were applied as treatments, their diameter increased significantly, to 0.20 ± 0.01, 0.23 ± 0.01, and 0.26 ± 0.01 μm, respectively, while, at the highest treatment concentration, it increased to 2.74 times that of the dexamethasone-induced control group (Figure 9C). In addition, when CAG (Figure 9B) was applied, the diameter of the myotubes increased in a concentration-dependent manner, increasing significantly at concentrations of 50 and 100 μg/mL to 0.25 ± 0.01 and 0.32 ± 0.01 μm, respectively (Figure 9D). At the highest treatment concentration, it increased to 2.86 times that of the dexamethasone-induced control group and was similar to that of the untreated control group.

Therefore, it was confirmed that UME and CAG significantly protect against dexamethasone-induced myotube atrophy.

#### 3.3.3. Expression of Atrogin-1, MuRF1, Myogenin, and MyoD1 Protein in DEX-Treated C2C12 Myotubes

MuRF1 and atrogin-1 act as ubiquitin E3 ligases and are expressed by the sub-signal system of GDF8, which involves smad 2/3. Additionally, they are stimulated by MAPK to enhance muscle protein degradation, contributing to muscle atrophy at the cellular level. This process is triggered by various environmental factors, such as increased glucocorticoid levels and reduced muscle usage. In summary, mechanisms of action involving Akt, mTOR, FoxO, etc., are directly associated with skeletal muscle atrophy and the synthesis and degradation of muscle proteins [42,43,44,45].

**Figure 10:**
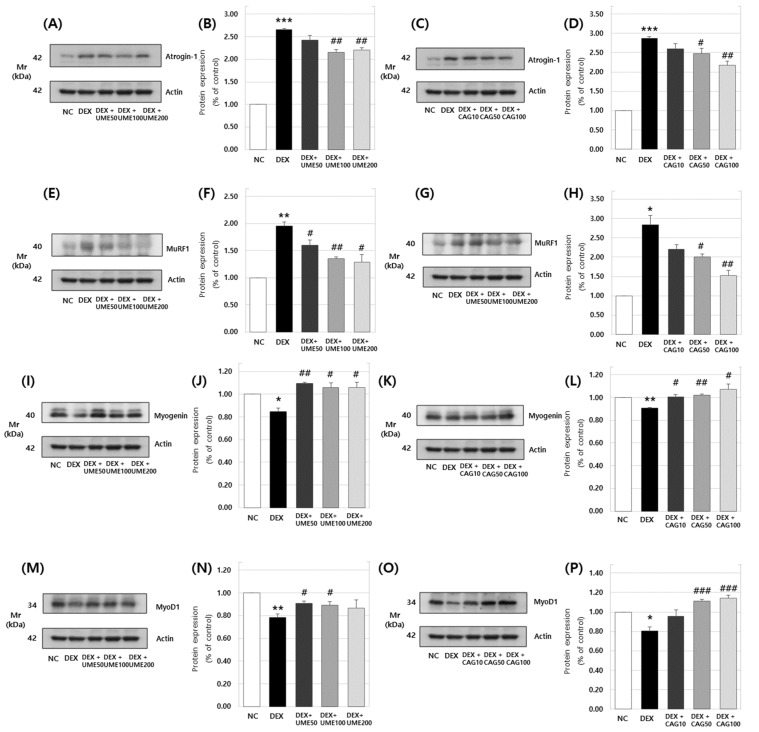
Effects of *Ulmus macrocarpa* extract (UME) and catechin 7-O-*β*-D apiofuranoside (CAG) on protein expression levels of (**A**–**D**) Atrogin-1, (**E**–**H**) MuRF1, (**I**–**L**) Myogenin, and (**M**–**P**) MyoD1 in DEX-treated C2C12 myotubes. UME and catechin 7-O-*β*-D were added to DEX-treated C2C12 myotubes and cultured for 24 h. Protein expression levels were determined using Western blot assay. Values are expressed as mean ± S.E.M. (n = 3). * *p* < 0.05, ** *p* < 0.01, and *** *p* < 0.001 are significantly different from that of the [(−)/(−)] group. ^#^ *p* < 0.05, ^##^ *p* < 0.01, and ^###^ *p* < 0.001 are significantly different from that of the [(+)/(−)] group.

The expression of Atrogin1 was investigated after UME (Figure 10A,B) and CAG (Figure 10C,D) treatments; the increased expression of Atrogin1 that was a result of the dexamethasone treatment decreased to 2.15 ± 0.07 and 2.21 ± 0.05 at concentrations of 100 and 200 μg/mL of UME, respectively. Treatment with CAG led to a significant decrease in Atrogin1 to 2.47 ± 0.13 and 2.17 ± 0.10 at concentrations of 50 μg/mL and 100 μg/mL, respectively. Additionally, the expression of MuRF1 increased to 1.96 ± 0.07 with the dexamethasone treatment alone, but decreased in a concentration-dependent manner when subsequently treated with UME (Figure 10E,F), falling to 1.35 ± 0.03 at a concentration of 100 μg/mL. Similarly, treatment with CAG (Figure 10G,H) led to a concentration-dependent decrease in MuRF1 expression, with significant decreases to 2.01 ± 0.07 and 1.52 ± 0.14 at concentrations of 50 μg/mL and 100 μg/mL, respectively.

On the other hand, the expression of Myogenin decreased to 0.84 ± 0.03 with the dexamethasone treatment alone but showed a concentration-dependent increase when subsequently treated with UME (Figure 10I,J), rising to 1.10 ± 0.01 at a concentration of 50 μg/mL. Treatment with CAG (Figure 10K,L) also led to a concentration-dependent increase in myogenin expression, particularly to 1.02 ± 0.01 at a concentration of 50 μg/mL, surpassing that of the untreated control group. The expression of MyoD1 decreased with the dexamethasone treatment alone, but showed a concentration-dependent increase when subsequently treated with UME (Figure 10M,N), reaching 0.91 ± 0.02 and 0.89 ± 0.03 at concentrations of 50 and 100 μg/mL, respectively. Similarly, treatment with CAG (Figure 10O,P) also led to a concentration-dependent increase, reaching 1.11 ± 0.02 and 1.14 ± 0.03 at concentrations of 50 and 100 μg/mL, surpassing that of the untreated control group (Figure 10). Therefore, it was confirmed that UME and CAG reduce the expression of Atrogin1 and MuRF1 while promoting the expression of myogenin and MyoD1.

#### 3.3.4. Effects on Muscle-Degradation- and Muscle-Synthesis-Related Gene Expression

To ensure the accuracy of this study, experiments were conducted on the expression of genes related to muscle degradation and synthesis. When treated with dexamethasone to induce muscle atrophy, the expression of the muscle-degradation-related genes Atrogin-1 mRNA and MuRF1 mRNA increased. When treated with 200 μg/mL of UME, the elevated expression of Atrogin-1 induced by dexamethasone decreased to 0.40 ± 0.04. Additionally, the expression of MuRF-1 significantly decreased at concentrations of 100 and 200 μg/mL to 0.56 ± 0.04 and 0.31 ± 0.06, respectively. However, the expression of MyoD and Myogenin, which are genes related to muscle synthesis, decreased when treated with dexamethasone and increased in a concentration-dependent manner when treated with UME. MyoD’s expression levels increased to 2.02 ± 0.26 and 2.26 ± 0.29 at UME concentrations of 50 and 100 μg/mL, respectively, while those of myogenin significantly increased to 1.51 ± 0.10 and 1.76 ± 0.17 (Table 2). 

In addition, when treated with CAG, the expression of Atrogin-1 and MuRF1 mRNA, which was increased by the dexamethasone treatment, showed a significant concentration-dependent decrease. Atrogin-1 decreased to 0.39 ± 0.04 and 0.16 ± 0.02 at 50 μg/mL and 100 μg/mL of CAG, and MuRF1 decreased to 0.45 ± 0.05 and 0.20 ± 0.03, respectively. The expression of MyoD1 mRNA increased to 1.56 ± 0.08 and 2.12 ± 0.13 at 50 μg/mL and 100 μg/mL, respectively. Myogenin’s mRNA expression showed a significant concentration-dependent increase, increasing significantly to 1.44 ± 0.14, 2.10 ± 0.17, and 2.26 ± 0.16 at each concentration (Table 3). As a result, it was confirmed that UME and CAG have a significant effect on inhibiting muscle degradation and promoting muscle synthesis by reducing the expression of Atrogin-1 and MuRF1 mRNA, which are muscle-degradation-related genes, and increasing the expression of MyoD and Myogenin, which are muscle-synthesis-related genes.

#### 3.3.5. Akt and mTOR Signaling Pathway-Related Muscle Protein Expression Investigation

Akt, also known as Protein kinase B, is known to phosphorylate various muscle proteins in skeletal muscles, influencing the growth and proliferation of muscle cells. The signaling of Akt, activated by IGF-1, has been reported to enhance muscle protein synthesis by suppressing the expression of apoptotic muscle proteins, such as caspase-9 [46]. When phosphorylation occurs in proteins like IGF-1 and Tuberous Sclerosis Complex 2 (TSC2), they become inactivated, translocating to the cytoplasm outside the nucleus and increasing the activity of mTOR [47]. mTOR is a crucial factor involved in the growth, metabolic action, and proliferation of cells, functioning as a serine/threonine protein kinase [48,49].

**Figure 11:**
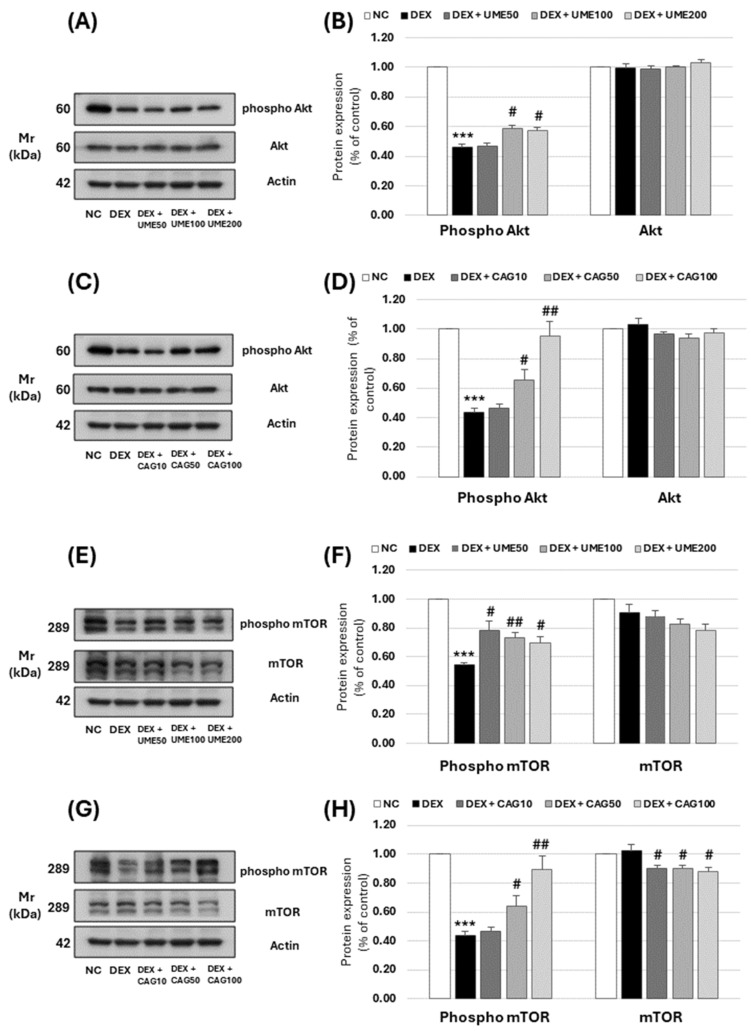
Effect of *Ulmus macrocarpa* extract (UME) and catechin 7-O-*β*-D apiofuranoside (CAG) on protein expression levels of (**A**–**D**) phospho-Akt and Akt in DEX-treated C2C12 myotubes and (**E**–**H**) phosphor-mTOR and mTOR in DEX-treated C2C12 myotubes. UME and CAG were added to DEX-treated C2C12 myotubes and cultured for 24 h, respectively. Protein expression levels were determined using Western blot assay. Values are expressed as mean ± S.E.M. (n = 3). *** *p* < 0.001 are significantly different from that of the [(−)/(−)] group. ^#^ *p* < 0.05, ^##^ *p* < 0.01 are significantly different from that of the [(+)/(−)] group.

The effect of UME and CAG on Akt signaling activity in dexamethasone-induced myotube cell atrophy was investigated (Figure 11). The expression of p-Akt significantly increased to 0.59 ± 0.02 and 0.58 ± 0.02 at UME (Figure 11A,B) concentrations of 100 and 200 μg/mL, respectively. When treated with CAG (Figure 11C,D), the highest treatment concentration of 100 μg/mL resulted in an increase to 0.95 ± 0.09, which was similar to that of the untreated control group.

Akt expression showed no significant difference with the addition of either UME or CAG. The expression of p-mTOR increased at all concentrations when treated with UME (Figure 11E,F), and was significantly increased to 0.73 ± 0.04 at 100 μg/mL. CAG (Figure 11G,H) led to a significant increase from 0.64 ± 0.07 at 50 μg/mL to 0.90 ± 0.09 at 100 μg/mL. The expression of mTOR did not differ significantly when treated with UME, but decreased when treated with CAG. These results confirmed that UME and CAG effectively promote muscle protein synthesis.

#### 3.3.6. FoxO Signaling Pathway-Related Muscle Protein Expression Investigation

The FoxO subgroup consists of FoxO1, FoxO3a, FoxO4, and FoxO6, which are located within muscles. This location protects the DNA-binding region called FoxO, which is a transcription factor located in the cell nucleus that regulates various muscle protein signaling. When FoxO is activated in the nucleus, it decomposes muscle proteins by regulating the ubiquitin proteasome and autophagy lysosome systems, which are muscle protein degradation pathways [47].

**Figure 12:**
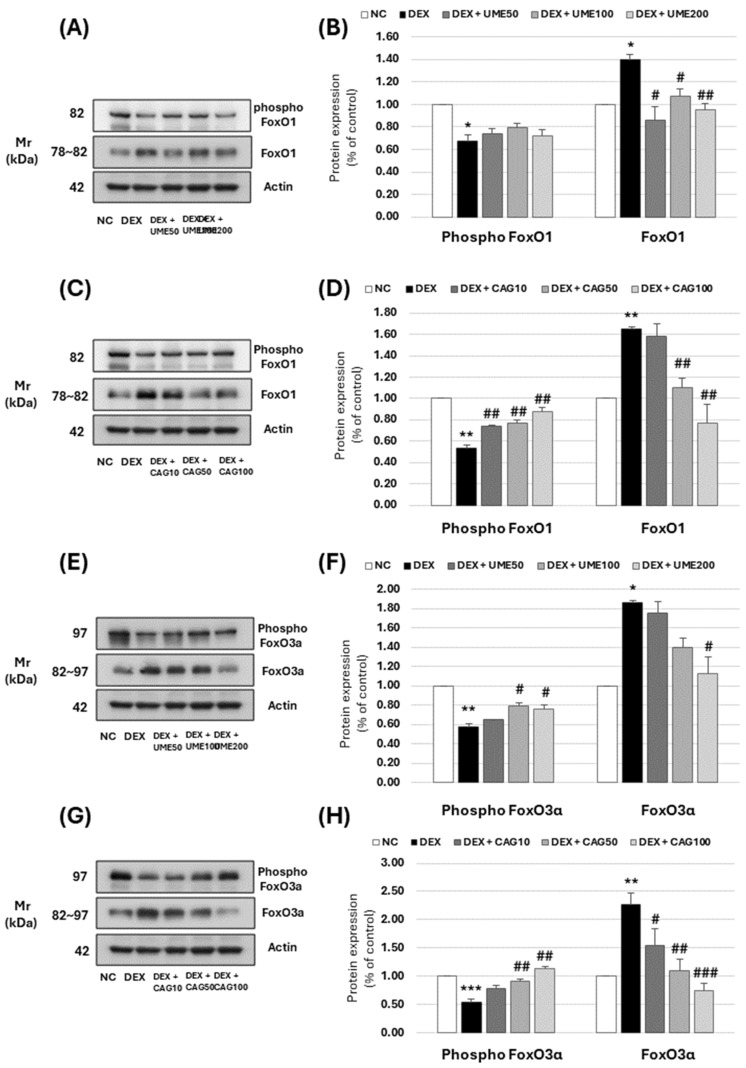
Effect of *Ulmus macrocarpa* extract (UME) and catechin 7-O-*β*-D apiofuranoside on protein expression levels of (**A**–**D**) phospho-FoxO1 and FoxO1 in DEX-treated C2C12 myotubes and (**E**–**H**) phospho-FoxO3a and FoxO3a in DEX-treated C2C12 myotubes. UME and CAG were added to DEX-treated C2C12 myotubes and cultured for 24 h, respectively. Protein expression levels were determined using Western blot assay. Values are expressed as mean ± S.E.M. (n = 3). * *p* < 0.05, ** *p* < 0.01, and *** *p* < 0.001 are significantly different from that of the [(−)/(−)] group. ^#^ *p* < 0.05, ^##^ *p* < 0.01, and ^###^ *p* < 0.001 are significantly different from that of the [(+)/(−)] group.

We examined the effects of UME and CAG on FoxO signaling activity in dexamethasone-induced myotube cell atrophy (Figure 12). The expression of phospho-FoxO1 increased in a concentration-dependent manner when treated with UME (Figure 12A,B) and significantly increased when treated with all concentrations of CAG (Figure 12C,D), 10, 50, and 100 μg/mL, rising to 0.74 ± 0.01, 0.77 ± 0.04, and 0.87 ± 0.04, respectively. The expression of FoxO1 also increased when treated with dexamethasone alone, but significantly decreased when treated with all concentrations of UME. In particular, at the highest treatment concentration of 200 μg/mL, it decreased to 0.95 ± 0.05, which was lower than that in the untreated control group. It was shown that the expression of phospho-FoxO3α decreased when treated with dexamethasone and significantly increased when treated with 100 μg/mL of UME (Figure 12E,F) or higher, leading to values of 0.79 ± 0.04 and 0.76 ± 0.04. When treated with CAG (Figure 12G,H), the expression of phospho-FoxO3α increased in a concentration-dependent manner, leading to a higher expression, especially at 100 μg/mL, than that seen in the untreated control group. The expression of FoxO3a significantly decreased to 1.12 ± 0.13 when treated with 200 μg/mL of UME. It was shown that treatment with CAG resulted in its decreased expression at all concentrations, particularly at 100 μg/mL, where the expression of FoxO3 was significantly reduced, to 0.75 ± 0.12, compared to the untreated control group. These results show that UME and CAG effectively inhibit the expression of muscle-degrading proteins (atrogin-1 and MuRF1).

## 4. Discussion

In existing research on plants belonging to the *Ulmus* genus, various outstanding biological activities have been reported, including antioxidant, anti-inflammatory, immune-modulating effects; the inhibition of angiogenesis; and the regulation of cell apoptosis in human papilla cells [5,11,12,50]. Plants of the *Ulmus* genus contain (-)-catechin, triterpene, and neolignan glycoside, and these are known to exert their effects through their me-tabolites after absorption and metabolism [5,6,7,8]. Many of the biological effects of *Ulmus* genus are believed to be mediated by its polyphenol catechins [51]. However, research on muscle loss and muscle atrophy is lacking. Therefore, our goal is to develop a therapeutic agent that utilizes natural compounds derived from *Ulmus macrocarpa* for the prevention of muscle loss and atrophy. Our research team conducted an in vitro mechanism study that covered four distinct categories—inhibiting oxidative stress, anti-apoptosis, promoting muscle protein synthesis, and inhibiting muscle protein degradation—to explore the effectiveness and mechanisms by which *Ulmus macrocarpa* prevents muscle loss and atrophy.

Reactive oxygen species (ROS) are formed within cells, generating free radicals that increase oxidative stress and trigger muscle cell apoptosis [52]. Bcl-2 family proteins play a crucial role in the inhibition and induction of cell apoptosis, with Bcl-2 acting as an inhibitor and Bax as an inducer [27]. The disruption of this balance leads to mitochondrial dysfunction, causing the release of cytochrome c from the inner mitochondrial membrane into the cytoplasm, stimulating various genes to induce cell apoptosis at low levels [32]. In H_2_O_2_-induced C2C12 cells, Bax was not significantly expressed, indicating that H_2_O_2_ does not influence Bax expression in these cells. However, treatment with catechin 7-O-*β*-D apiofuranoside significantly increased the expression of Bcl-2. These findings suggest that catechin 7-O-*β*-D apiofuranoside prevents the loss of mitochondrial function due to ROS in H_2_O_2_-treated C2C12 cells, inhibiting the activation of the apoptosis pathway and enhancing cell viability. Our results demonstrate that the increased expression of Bcl-2 in C2C12 cells treated with catechin 7-O-*β*-D apiofuranoside could potentially block mitochondrial dysfunction, thereby suppressing the induction of cell apoptosis. Additionally, caspase-9, a key player in the induction of cell apoptosis, is presumed to induce the activity of caspases such as caspase-3, leading to the expression of PARP and DNA fragmentation [34]. PARP plays a crucial role in maintaining DNA repair and genetic stability in normal cells. Our results also indicate that catechin 7-O-*β*-D apiofuranoside inhibits the expression of caspase-3 and PARP induced by H_2_O_2_ in C2C12 cells. This outcome highlights the inhibitory effect of catechin 7-O-*β*-D apiofuranoside on the induction of caspase-3 and PARP in response to H_2_O_2_ treatments.

Myogenesis becomes imperative when muscle regeneration is required due to the reduced muscle mass caused by atrophy. It involves the differentiation of cells into myotubes, regulated by myogenic regulatory factors (MRFs) such as myogenin and myoD [53,54]. Atrogin-1 and MuRF1 are recognized as ubiquitin–proteasome protein degradation factors contributing to muscle atrophy [42,55]. Dexamethasone (DEX), a prominent glucocorticoid, induces skeletal muscle degradation when inappropriately used in clinical settings, making it a widely employed agent to simulate muscle cell atrophy in in vitro systems [35,36]. To assess the impact of *Ulmus macrocarpa* extract and catechin 7-O-*β*-D apiofuranoside on muscle degradation and synthesis in a dexamethasone-induced muscle atrophy model, this study investigated the protein and gene expression of key biomarkers: myoD, myogenin, atrogin-1, and MuRF1. Treatment with *Ulmus macrocarpa* extract or catechin 7-O-*β*-D apiofuranoside resulted in the decreased expression of atrogin-1 and MuRF1, while the expression of myogenin and myoD increased. This confirmed that catechin 7-O-*β*-D apiofuranoside mitigates muscle atrophy by downregulating muscle proteins and genes involved in the muscle synthesis pathway, thereby promoting muscle differentiation. Muscle protein synthesis is facilitated through the insulin-like growth factor 1/PI3K/Akt signaling pathway, while muscle protein degradation is governed by a signaling pathway comprising forkhead box O (FoxO) and the ubiquitin–proteasome system [56]. Catechin 7-O-*β*-D apiofuranoside was found to inhibit muscle atrophy through the Akt/FoxO signaling pathway. Fluorescent staining under a microscope revealed the inhibitory effect of catechin 7-O-*β*-D apiofuranoside on muscle atrophy, with myotube cell diameters reduced by dexamethasone and returning to normal post-treatment. This observation confirmed that the restoration was to a level nearly identical to that of the control group.

In conclusion, this study demonstrates that *Ulmus macrocarpa* extract, including catechin compounds known to have antioxidant, anti-diabetic [57], and anti-obesity effects [58], can prevent and inhibit muscle loss and atrophy. In addition, since the muscle loss prevention effect of *Ulmus macrocarpa* extract was verified in a recent in vivo experiment [10], it is thought that *Ulmus macrocarpa* extract can be utilized as a key ingredient in the development of functional foods and health supplements for preventing muscle loss and atrophy and sarcopenic obesity in diabetic patients in the future.

## Figures and Tables

**Table 1:** Specific primer sequences for PCR.

mRNA	Primer Sequences	
Atrogin-1	Forward	5′-GCCCTCCACACTAGTTGACC-3′
	Reverse	5′-GACGGATTGACAGCCAGGAA-3′
MuRf-1	Forward	5′-GAGGGCCATTGACTTTGGGA-3′
	Reverse	5′-TTTACCCTCTGTGGTCACGC-3′
MyoD1	Forward	5′-GCACTACAGTGGCGACTCAGAT-3′
	Reverse	5′-TAGTAGGCGGTGTCGTAGCCAT-3′
Myogenin	Forward	5′-CCATCCAGTACATTGAGCGCCT-3′
	Reverse	5′-CTGTGGGAGTTGCATTCACTGG-3′
GAPDH	Forward	5′-TGGGTGTGAACCATGAGAAG-3′
	Reverse	5′-GCTAAGCAGTTGGTGGTGC-3′

**Table 2:** Effect of *Ulmus macrocarpa* extract on muscle-degradation- and muscle-synthesis-related gene expression.

DEX (5 μM)	UME (μg/mL)	mRNA			
		Atrogin-1	MuRF-1	Myo D	Myogenin
−	−	0.07 ± 0.02	0.10 ± 0.18	3.24 ± 0.27	2.47 ± 0.24
+	−	1.00 ± 0.22 **	1.00 ± 0.10 ***	1.00 ± 0.11 ***	1.00 ± 0.07 ***
+	50	1.03 ± 0.12	0.90 ± 0.06	1.47 ± 0.21	1.27 ± 0.09 ^#^
+	100	0.60 ± 0.04	0.56 ± 0.04 ^##^	2.02 ± 0.26 ^##^	1.51 ± 0.10 ^##^
+	200	0.40 ± 0.04 ^#^	0.31 ± 0.06 ^###^	2.26 ± 0.29 ^##^	1.76 ±0.17 ^##^

DEX: dexamethasone; UME: *Ulmus macrocarpa* extract. Values are expressed as mean ± S.E.M. (n = 3). The target mRNA’s expression was normalized to that of GAPDH. ** *p* < 0.01, *** *p* < 0.001 are significantly different from that of the [(−)/(−)] group. ^#^ *p* < 0.05, ^##^ *p* < 0.01, and ^###^ *p* < 0.001 are significantly different from that of the [(+)/(−)] group.

**Table 3:** Effect of catechin 7-O-*β*-D apiofuranoside on the expression of muscle-degradation- and muscle-synthesis-related genes.

DEX (5 μM)	CAG (μg/mL)	mRNA			
		Atrogin-1	MuRF-1	Myo D	Myogenin
−	−	0.06 ± 0.01	0.11 ± 0.02	2.98 ± 0.29	3.34 ± 0.55
+	−	1.00 ± 0.16 **	1.00 ± 0.08 ***	1.00 ± 0.09 ***	1.00 ± 0.10 **
+	10	0.75 ± 0.05	0.75 ± 0.06 ^#^	1.16 ± 0.12	1.44 ± 0.14 ^#^
+	50	0.39 ± 0.04 ^##^	0.45 ± 0.05 ^###^	1.56 ± 0.08 ^##^	2.10 ± 0.17 ^##^
+	100	0.16 ± 0.02 ^###^	0.20 ± 0.03 ^###^	2.12 ± 0.13 ^###^	2.26 ±0.16 ^###^

DEX: dexamethasone. CAG: Catechin 7-O-*β*-D apiofuranoside. Values are expressed as mean ± S.E.M. (n = 3). The target mRNA’s expression was normalized to that of GAPDH. ** *p* < 0.01, *** *p* < 0.001 are significantly different from that of the [(−)/(−)] group. ^#^ *p* < 0.05, ^##^ *p* < 0.01, and ^###^ *p* < 0.001 are significantly different from that of the [(+)/(−)] group.

## Data Availability

The original contributions presented in the study are included in the article, further inquiries can be directed to the corresponding author.

## Abbreviations

Akt	protein kinase B
Bax	bcl-2-associated X protein
Bcl-2	B-cell leukemia/lymphoma 2 protein
CAG	catechin 7-O-*β*-D apiofuranoside
DAPI	4′, 6-diamidino-2-phenylindole
DEX	dexamethasone
FoxO	forkhead box O
HPLC	high-performance liquid chromatography
mTOR	mammalian target of rapamycin
MuRF1	muscle RING-finger protein-1
MyoD	myoblast determination
PARP	Poly (ADP-ribose) polymerases
RT-PCR	real-time polymerase chain reaction
UME	*Ulmus macrocarpa* extract
